# Therapeutic Drug Monitoring of Direct Oral Anticoagulants and Its Association with Clinical Outcomes: A Systematic Review and Meta-Analysis

**DOI:** 10.3390/ph19020215

**Published:** 2026-01-26

**Authors:** Layaly Bakir, Ibrahim Mohamed, Sharoma Yesukumar, Rasha Abduljabbar, Ibrahim Yusuf Abubeker, Mohammed I. Danjuma

**Affiliations:** 1 Internal Medicine Residency Program, Division of General Medicine, Hamad Medical Corporation, Doha P.O. Box 3050, Qatar; 2 Internal Medicine Department, Hamad General Hospital, Doha P.O. Box 3050, Qatar; 3 Medical School, University of Nottingham, Nottingham NG7 2UH, UK; 4 Mayo Clinic Rochester, Rochester, MN 55905, USA; 5 College of Medicine, QU Health, Qatar University, Doha P.O. Box 2713, Qatar; 6 Weill Cornell College of Medicine, Doha P.O. Box 24144, Qatar

**Keywords:** DOAC, therapeutic drug monitoring, meta-analysis, anticoagulation, thrombosis, bleeding

## Abstract

**Background:** Direct oral anticoagulants (DOACs) are now the preferred anticoagulant over vitamin K antagonists for patients with atrial fibrillation (AF) and venous thromboembolism (VTE). Variability in drug exposure raises concerns about bleeding and thrombotic events, highlighting the potential value of therapeutic drug monitoring (TDM). **Methods:** This systematic review and meta-analysis conducted a systematic search of PubMed, Embase, Web of Science, Scopus, Cochrane Library, and ClinicalTrials.gov (from inception to May 2025) and identified studies reporting DOAC levels and clinical outcomes. Two reviewers independently performed screening, data extraction, and risk-of-bias assessment (RoB 2.0, Newcastle–Ottawa Scale). Random-effects meta-analytical models generated pooled estimates, with meta-regression exploring potential sources of variability (DOAC type, drug levels) and exposure–response relationships. **Results:** Nineteen studies comprising 5770 patients were included in the review. The pooled event rates were 8% for major bleeding (95% CI: 0.05–0.11), 7% for thrombotic events (95% CI: 0.05–0.09), and 3% for mortality (95% CI: 0.03–0.04). Heterogeneity was substantial for bleeding and thrombotic events (*I*^2^ = 95.6% and 87.3%, respectively) but negligible for mortality (*I*^2^ = 0%). Meta-regression analyses showed no significant association between mean DOAC concentration and either major bleeding (β = −0.00021, *p* = 0.35, Adj R^2^ ≈ 0%) or thrombotic events (β = 0.00005, *p* = 0.78, Adj R^2^ ≈ 0%), indicating that variations in measured plasma levels did not meaningfully explain event rate differences across studies. **Conclusions:** In this systematic review and meta-analysis, measured DOAC concentrations show limited and inconsistent association with clinical outcomes. While the present synthesis does not demonstrate a statistically robust linear correlation between DOAC plasma concentrations and adverse outcomes, it highlights the multifactorial determinants of bleeding and thrombosis risk underscores the potential value of selective TDM in individualized care. Further prospective, standardized studies are needed to define clinically actionable thresholds and to validate TDM-guided strategies that optimize the delicate balance between safety and efficacy in DOAC therapy.

## 1. Introduction

Direct oral anticoagulants (DOACs) such as dabigatran, rivaroxaban, apixaban, and Edoxaban, have become the preferred agents for stroke and systemic embolization prevention in non-valvular atrial fibrillation and the treatment and prevention of recurrent venous thromboembolism [1,2,3,4,5,6]. This is due to their predictable pharmacokinetics and fixed dosing regimens with lower burden of drug–drug interactions compared to vitamin K antagonists [1,2]. Despite their advantages, concerns remain regarding the optimal management of patients with extreme body weights, renal impairment, elderly patients, and those on interacting medications [7,8,9]. Variability in plasma concentrations among patients receiving DOACs can lead to significant differences in anticoagulant efficacy and safety, potentially increasing the risk of bleeding and thrombotic complications [10,11]. Therapeutic drug monitoring (TDM) refers to determination of specific drug concentrations in a patient’s serum at designated intervals to maintain a constant plasma drug concentration with the view to optimizing individual therapeutic effectiveness and minimizing adverse events [12]. While TDM has been standard practice for drugs with narrow therapeutic indices (such as warfarin, Digoxin, its routine implementation for DOACs therapeutics remains controversial. This stems from the perceived predictability of their pharmacokinetics in most populations [13]. However, recent evidence suggests significant pharmacokinetic variability among individuals taking DOACs, particularly in specific high-risk patient groups [7,14,15,16,17,18]. Recent pharmacokinetic studies have consistently reported clinically significant inter- and intra-individual variability in DOAC plasma concentrations, particularly among patients with renal dysfunction or extremes of body weight [19,20]. Despite these findings, earlier literature stopped short of conclusively establishing a direct link between measured plasma levels of DOACs and key clinical endpoints such as thrombotic events and major bleeding [21]. Clinical guidelines from leading organizations, including the International Society on Thrombosis and Haemostasis (ISTH) and the European Heart Rhythm Association, currently do not advocate routine DOAC monitoring, primarily due to the lack of evidence from large-scale randomized controlled trials [15,22,23]. Nonetheless, selective TDM in specific patient populations, such as elderly individuals, patients with significant renal impairment, or those receiving concurrent medications that affect DOAC metabolism, has been proposed to mitigate risks associated with pharmacokinetic variability [24]. In the context of DOACs, routine TDM has not been adopted due to their relatively stable pharmacokinetic profiles in most patients. Yet emerging evidence suggests that clinically significant pharmacokinetic variability does occur, especially in high-risk subgroups. A recent systematic review by de Vries et al. (2025) compiled “usual on-therapy” DOAC concentration ranges in AF patients and found a broad 3- to 5-fold inter-individual spread in drug levels (For example, for apixaban 5 mg twice daily, reported trough concentrations (10th–90th percentile) ranged from ~58 to 206 ng/mL, and similarly wide ranges were seen for other DOACs [25]. This variability underscores that despite fixed dosing, one patient’s “on-therapy” level can be several-fold higher or lower than another’s [25]. Such differences may be especially pronounced in patients with renal dysfunction or extreme body size, who were often excluded from pivotal trials. Given this uncertainty and the potentially severe consequences of subtherapeutic or supratherapeutic drug levels, systematically synthesizing existing literature to quantify the association between DOAC plasma concentrations and clinical outcomes becomes essential. The current systematic review and meta-analysis aim to critically evaluate the relationship between measured plasma concentrations of DOACs and adverse clinical events, specifically focusing on bleeding and thrombotic complications. By quantitatively pooling available data, this review intends to clarify the clinical relevance of DOAC TDM, identify high-risk patient groups most likely to benefit from monitoring, and inform future clinical guidelines and practices.

## 2. Methods

### 2.1. Protocol and Registration

Protocol registered with PROSPERO (CRD420251108204); we followed PRISMA 2020 guidelines in the conduct of the analyses [26].

### 2.2. Eligibility Criteria

We included studies enrolling adult patients (≥18 years) receiving any direct oral anticoagulant (DOAC) with marketing authorization—including dabigatran, rivaroxaban, apixaban, or Edoxaban for all indications including atrial fibrillation (AF), venous thromboembolism (VTE), or other regulatory-approved clinical indications. Eligible studies were required to have had quantitatively measured DOAC plasma concentrations, either at peak (*Cmax*) or trough (*Cmin*) levels, using all validated laboratory methods (e.g., liquid chromatography–tandem mass spectrometry, chromogenic anti-Xa assays, diluted thrombin time). Additionally, studies need to report at least one hard clinical outcome of interest: bleeding events (major, clinically relevant non-major, or any bleeding as defined by the study) or thromboembolic events (ischemic stroke, systemic embolism, recurrent VTE). We considered randomized controlled trials (RCTs), prospective or retrospective cohort studies, and case–control studies eligible for inclusion. Case series, narrative reviews, editorials, conference abstracts without sufficient data, and studies reporting only surrogate laboratory endpoints without clinical outcomes were excluded.

### 2.3. Information Sources and Search Strategy

A comprehensive literature search was conducted through PubMed, Embase, Scopus, Cochrane Central Register of Controlled Trials (CENTRAL), Web of Science, and ClinicalTrials.gov from database inception to 31 May 2025. We used a combination of controlled vocabulary (e.g., MeSH, Emtree) and free-text search terms, including: “direct oral anticoagulants” OR “DOAC” OR “dabigatran” OR “rivaroxaban” OR “apixaban” OR “Edoxaban” AND “plasma concentration” OR “drug monitoring” OR “therapeutic drug monitoring” OR “TDM” OR “pharmacokinetics” AND “bleeding” OR “hemorrhage” OR “thrombosis” OR “stroke” OR “venous thromboembolism”. The search strategy was tailored for each database, with Boolean operators and proximity searches where applicable. No language restrictions were applied. The reference lists of all included studies and relevant reviews were also manually screened to identify additional eligible titles and abstracts (Supplementary Table S1).

### 2.4. Study Selection

Titles and abstracts identified from the search were independently screened by two reviewers (LB and IM) using Rayyan QCRI systematic review software 2016. The reviewers applied the eligibility criteria to each record, classifying them as “include,” “exclude,” or “maybe.” Full-text articles were then retrieved for all studies deemed potentially eligible. Any disagreements at either screening stage were resolved through discussion, and when consensus could not be reached, a third reviewer (SY) adjudicated the decision. The study selection process will be presented using a PRISMA 2020 flow diagram.

### 2.5. Data Extraction

Data from the included studies were extracted into a standardized, pilot-tested data extraction form. The following variables were collected: study characteristics (year of publication, country, study design, follow-up duration); population characteristics (age, sex distribution, comorbidities, renal function categories, body weight/BMI); intervention details (DOAC type, dose regimen, indication, concomitant medications); TDM details (assay methodology, calibration standards, sample timing, definition of therapeutic or supra/subtherapeutic thresholds); clinical outcomes (bleeding events, thromboembolic events, mortality); and effect estimates (unadjusted and adjusted odds ratios, risk ratios, hazard ratios, and 95% confidence intervals). Where multiple publications from the same cohort were identified, we extracted the most complete and updated dataset.

### 2.6. Risk of Bias Assessment

The methodological quality of the included studies was evaluated using the Newcastle–Ottawa Scale (NOS), which is specifically designed for assessing non-randomized studies such as observational cohorts and cross-sectional investigations. This tool was chosen because most studies included in this meta-analysis were retrospective or prospective cohorts without randomization, making NOS the most appropriate framework for evaluating selection bias, comparability of groups, and outcome ascertainment. Each study was appraised across three domains; selection, comparability, and outcome with a maximum possible score of nine points. Domain-specific judgments were expressed as Low, Unclear, High, or Critical risk of bias, and overall ratings were derived based on the cumulative NOS score (≥8 = Low; 6–7 = Moderate; 4–5 = High; ≤3 = Critical). The resulting dataset was formatted in a ROBVIS-compatible structure and visualized using the ROBVIS web tool, which generated both study-level traffic-light plots and summary bar charts to illustrate the distribution of bias across domains. Assessments were conducted independently by two reviewers (MID and LB), and discrepancies were resolved through discussion with a third reviewer (IM).

## 3. Outcomes Definition

Given the pooling of studies from different study/trial designs it is inevitable that differences in clinical outcome definitions may occur. We extracted outcome definitions exactly as stated from each study and harmonized to ISTH criteria wherever possible. Major bleeding, clinically relevant non-major bleeding (CRNMB), and thromboembolic outcomes were mapped to ISTH-equivalent categories as appropriate. Variability across definitions was handled using random-effects modeling with inverse variance weighting.

### Data Synthesis

We conducted a random-effects meta-analysis using the DerSimonian–Laird method to pool ORs or RRs across studies, separately for bleeding and thromboembolic outcomes. Where adjusted estimates were available, they were prioritized over unadjusted measures. Statistical heterogeneity was assessed using the *I*^2^ statistic (values >50% indicating substantial heterogeneity) and Cochran’s Q test (*p* < 0.10 considered significant). Pre-specified subgroup analyses included DOAC type (Dabigatran, Rivaroxaban, Apixaban, Edoxaban), and sample timing (peak vs. trough concentration). Sensitivity analyses were performed excluding studies at high risk of bias and using leave-one-out methods. Publication bias was evaluated for outcomes with ≥3 studies using funnel plots and Egger’s regression test. All analyses were carried out with Stata (StataCorp. Stata Statistical Software: Release 15, College Station, TX, USA, StataCorp LLC; 2017).

## 4. Results

### 4.1. Search Results

**Scheme 1:**
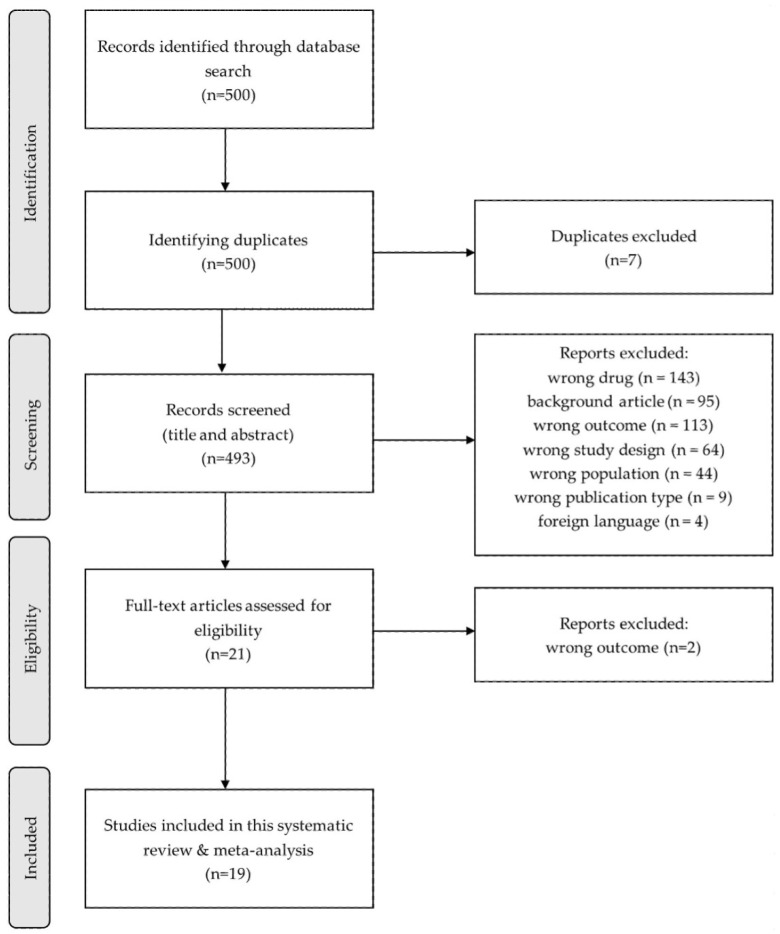
PRISMA flow diagram.

A comprehensive literature search was conducted using PubMed, Embase, Scopus, Cochrane Library, Web of Science, and ClinicalTrials.gov, generating 500 initial records. After deduplication, titles and abstracts were screened, resulting in 21 articles retrieved for full-text evaluation. Following a detailed assessment, 19 studies encompassing 5770 patients were deemed eligible and included in the final meta-analysis. The PRISMA flow diagram (Scheme 1) outlines this selection process.

### 4.2. Study Characteristics

Details of the included studies including socio-demographics characteristics are summarized in Table 1. The studies were published between 2018 and 2025 and collectively enrolled 5770 participants. The mean age ranged from 41.2 to 80 years, representing an older population typical of individuals treated with direct oral anticoagulants (DOACs). Male participants accounted for approximately 45.9% to 64.1% of study cohorts. The primary treatment indication across the studies was atrial fibrillation (84%), while a smaller proportion of studies included patients treated for venous thromboembolism (57.8%), including deep vein thrombosis (54.4%) and pulmonary embolism (54.4%). Most studies were either retrospective or prospective in design, with a few descriptive analyses. Sample sizes ranged from 60 to 1657 patients. The DOAC agents assessed included Apixaban (84.2%), Rivaroxaban (78.9%), Dabigatran (47.3%), and Edoxaban (21%). Therapeutic drug monitoring (TDM) was performed using anti-Factor Xa chromogenic assays (68.4%) or liquid chromatography–tandem mass spectrometry (LC–MS/MS) (31.5%), though thresholds for defining therapeutic levels varied substantially between studies. Major bleeding outcomes were reported in 15 studies and thrombotic events were evaluated in 12 studies.

### 4.3. Risk of Bias

**Scheme 2:**
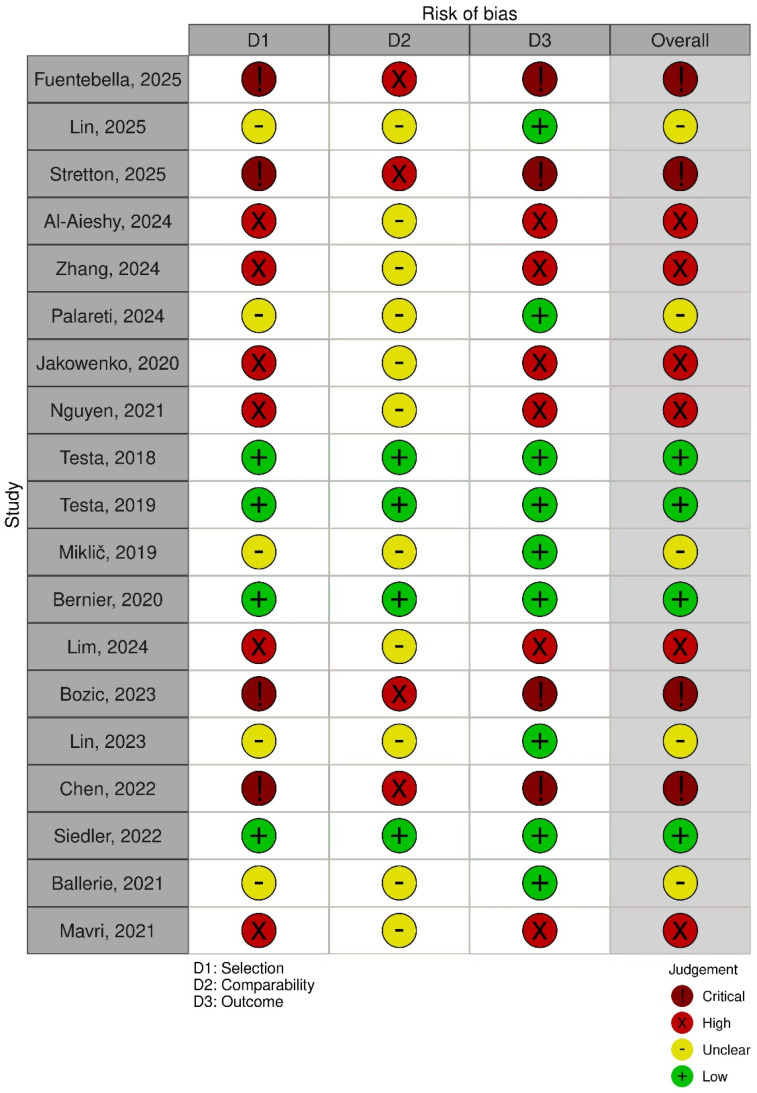
Risk of Bias Summary (Newcastle–Ottawa Scale) Traffic-light plot generated using the ROBVIS tool illustrating domain-specific and overall risk-of-bias judgments for all included studies (*n* = 19). D1 = Selection; D2 = Comparability; D3 = Outcome. Green (+) = Low risk of bias; Yellow (−) = Unclear risk; Red (X) = High risk; Red (!) = Critical risk. Judgments reflect reviewer consensus based on the Newcastle–Ottawa Scale, where higher risk categories denote greater methodological limitations related to study design, confounding, or outcome ascertainment. (Jakowenko, 2020 [10]; Nguyen, 2021 [8]; Testa, 2018 [11]; Testa, 2019 [20]; Miklič, 2019 [13]; Bernier, 2020 [12]; Fuentebella, 2025 [5]; Lin, 2025 [4]; Stretton, 2025 [6]; Al-Aieshy, 2024 [7]; Zhang, 2024 [15]; Palareti, 2024 [14]; Lim, 2024 [18]; Bozic, 2023 [16]; Lin, 2023 [2]; Chen, 2022 [17]; Siedler, 2022 [3]; Ballerie, 2021 [1]; Mavri, 2021 [9].)

Supplementary Table S2 outlines the methodological quality and risk of bias of the 19 included studies, assessed using the Newcastle–Ottawa Scale (NOS). Scores ranged from 3 to 8 out of a possible 9 points. High-quality studies, such as those by Testa et al. [11,20] and Bernier et al., 2020 [12], achieved scores of 8, reflecting strong study design and execution. Nearly half of the studies (47%) were classified as having low or moderate risk of bias, indicating generally sound methodologies despite limitations typical of real-world data, such as retrospective designs and single-center settings. These assessments capture a broad range of patient populations and clinical contexts, thereby enhancing the depth and applicability of our meta-analysis. Visual representation of risk of bias is shown in Scheme 2.

### 4.4. Meta-Analysis Outcomes

#### 4.4.1. Overall Major Bleeding Rate

**Figure 1:**
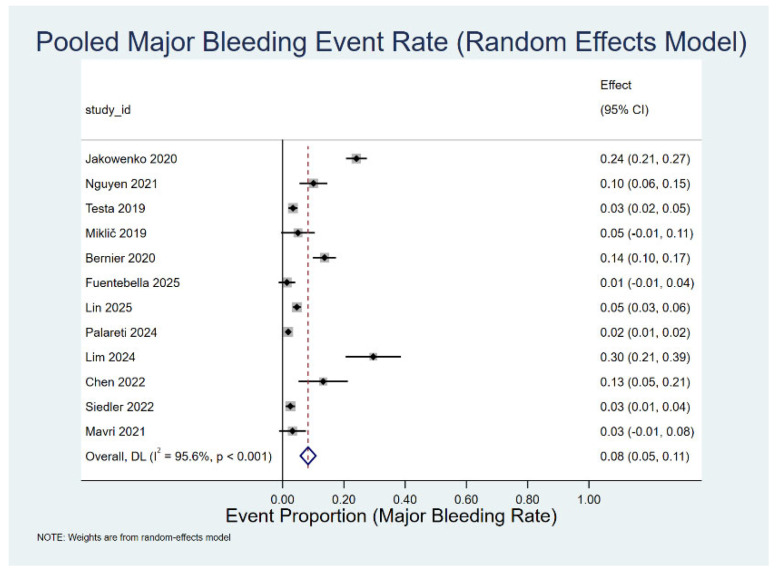
**Pooled Major Bleeding Event Rate (Random-Effects Model)**. Forest plot showing individual study proportions and 95% confidence intervals (CIs) for major bleeding events among patients on direct oral anticoagulants (DOACs). The pooled estimate (diamond) represents a major bleeding rate of **8% (95% CI: 0.05–0.11)** with substantial heterogeneity (**I^2^ = 95.6%, *p* < 0.001**). Weights are from a random-effects (DerSimonian–Laird, Freeman–Tukey transformation) model. The black squares represent individual study event proportions, with square size proportional to study weight; horizontal lines indicate 95% confidence intervals. The vertical dashed line denotes the pooled estimate, and the blue diamond represents the overall random-effects summary with its 95% confidence interval. (Jakowenko, 2020 [10]; Nguyen, 2021 [8]; Testa, 2019 [20]; Miklič, 2019 [13]; Bernier, 2020 [12]; Fuentebella, 2025 [5]; Lin, 2025 [4]; Palareti, 2024 [14]; Lim, 2024 [18]; Chen, 2022 [17]; Siedler, 2022 [3]; Mavri, 2021 [9]).

The pooled rate of major bleeding across all included studies was 8% (95% CI: 0.05–0.11), with substantial heterogeneity (*I*^2^ = 95.6%, *p* < 0.001) (Figure 1). The highest bleeding risks were reported in Lim 2024 [18] (30%, 95% CI: 0.21–0.39), Jakowenko 2020 [10] (24%, 95% CI: 0.21–0.27), and Bernier 2020 [12] (14%, 95% CI: 0.10–0.17). In contrast, the lowest risks were observed in Fuentebella 2025 [5] (1%, 95% CI: −0.01–0.04), Palareti 2024 [14] (2%, 95% CI: 0.01–0.02), and Testa 2019 [20] (3%, 95% CI: 0.02–0.05).

#### 4.4.2. Effect of Assay Methodology on Clinical Outcomes

**Figure 2:**
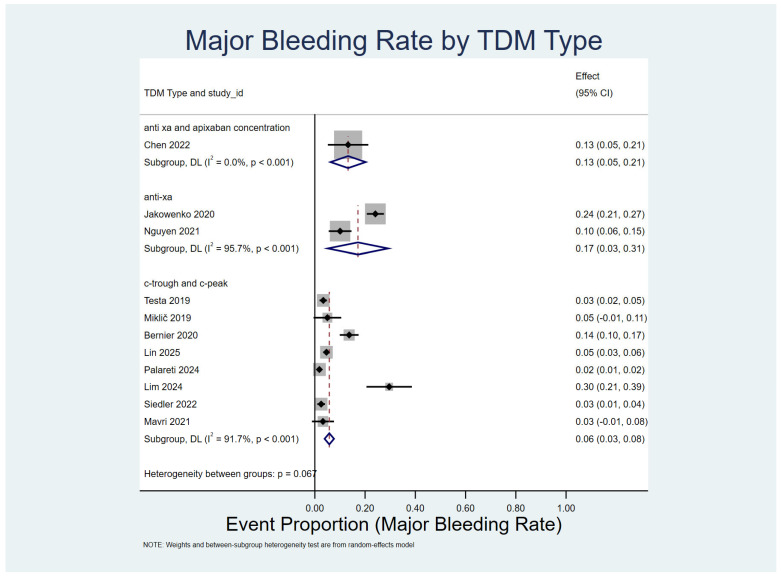
**Major Bleeding Rate by Therapeutic Drug Monitoring (TDM) Type**. Subgroup forest plot stratified by assay methodology used for DOAC concentration measurement. Pooled bleeding rates were **13% (95% CI: 0.05–0.21)** for anti-Xa/apixaban assays, **17% (95% CI: 0.03–0.31)** for anti-Xa only, and **6% (95% CI: 0.03–0.08)** for trough/peak measurements. Between-group heterogeneity approached statistical significance (***p* = 0.067**), indicating variability across assay types. (Jakowenko, 2020 [10]; Nguyen, 2021 [8]; Testa, 2019 [20]; Miklič, 2019 [13]; Bernier, 2020 [12]; Lin, 2025 [4]; Palareti, 2024 [14]; Lim, 2024 [18]; Chen, 2022 [17]; Siedler, 2022 [3]; Mavri, 2021 [9]).

Subgroup analyses according to assay methodology demonstrated variability. For studies using anti–Factor Xa assays, the pooled bleeding rate was 17% (95% CI: 0.03–0.31, *I*^2^ = 95.7%), with Jakowenko 2020 [10] (24%) and Nguyen 2021 [8] (10%) contributing most to the pooled outcome (Figure 2). In contrast, studies using peak/trough concentration measurements reported a pooled bleeding rate of 6% (95% CI: 0.03–0.08, *I*^2^ = 91.7%), though this finding was largely influenced by Lim 2024 [18] (30%). Only a single study, Chen 2022 [17], reported outcomes using combined anti–Xa and apixaban-specific assays, with a bleeding rate of 13% (95% CI: 0.05–0.21). Between-group heterogeneity approached statistical significance (*p* = 0.067), indicating variability across assay types.

#### 4.4.3. Major Bleeding by DOAC Indication (AF vs. VTE)

**Figure 3:**
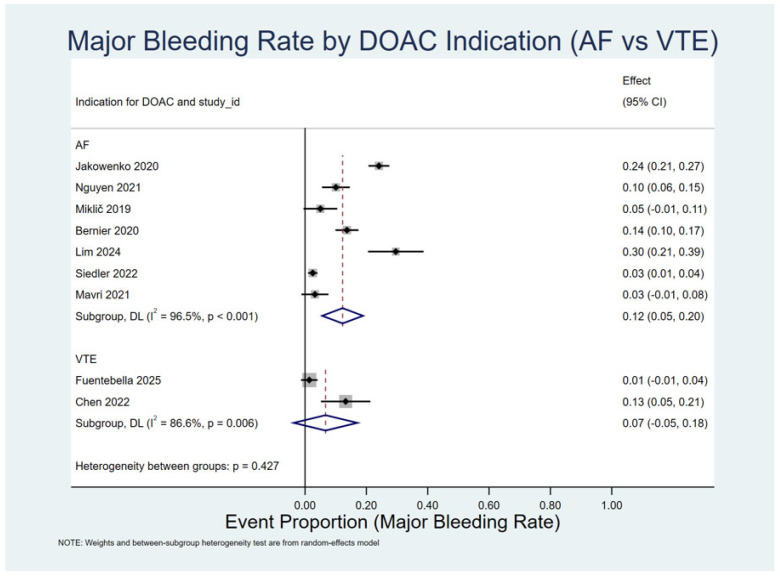
**Major Bleeding Rate by DOAC Indication (Atrial Fibrillation vs. Venous Thromboembolism)**. Subgroup forest plot comparing major bleeding event rates between studies enrolling patients with atrial fibrillation (AF) and venous thromboembolism (VTE). The pooled rate for AF studies was **12% (95% CI: 0.05–0.20; I^2^ = 96.5%)**, and for VTE studies **7% (95% CI: –0.05 to 0.18; I^2^ = 86.6%)**. Between-group heterogeneity was not statistically significant (***p* = 0.43**). (Jakowenko, 2020 [10]; Nguyen, 2021 [8]; Miklič, 2019 [13]; Bernier, 2020 [12]; Lim, 2024 [18]; Siedler, 2022 [3]; Mavri, 2021 [9]; Fuentebella, 2025 [5]; Chen, 2022 [17]).

When analyzed by clinical indication, patients with atrial fibrillation (AF) had a pooled major bleeding rate of 12% (95% CI: 0.05–0.20, *I*^2^ = 96.5%), with the highest risks again reported in Lim 2024 [18] (30%) and Jakowenko 2020 [10] (24%) (Figure 3). In patients treated for venous thromboembolism (VTE), the pooled bleeding rate was 7% (95% CI: −0.05–0.18, *I*^2^ = 86.6%), with rates ranging from 1% in Fuentebella 2025 [5] to 13% in Chen 2022 [17]. No significant difference was observed between AF and VTE subgroups (*p* = 0.43).

#### 4.4.4. Overall Thrombotic Event Rate

**Figure 4:**
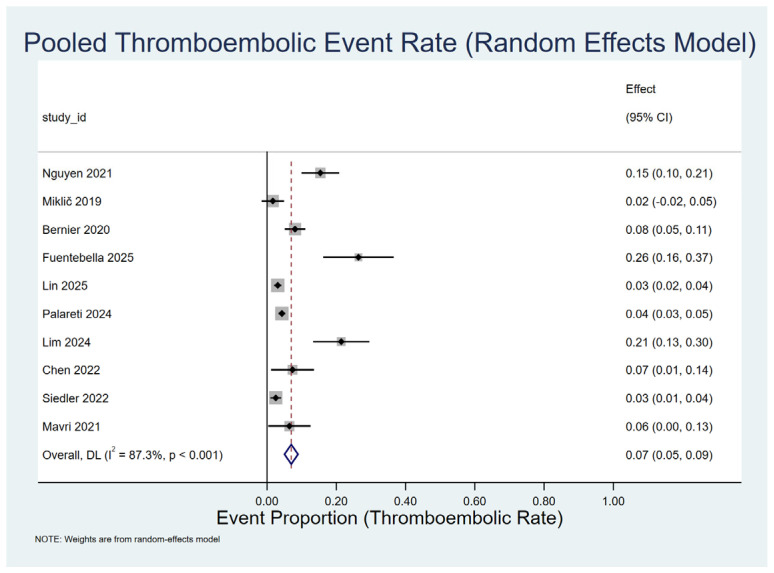
**Pooled Thromboembolic Event Rate (Random-Effects Model)**. Forest plot showing individual study and pooled thrombotic event proportions. The pooled rate was **7% (95% CI: 0.05–0.09)** with high heterogeneity (**I^2^ = 87.3%, *p* < 0.001**). Highest event rates were observed in **Fuentebella 2025** [5] **(26%)** and **Lim 2024** [18] **(21%)**, while the lowest were **Lin 2025** [4] **(3%)** and **Palareti 2024** [14] **(4%)**. (Nguyen, 2021 [8]; Miklič, 2019 [13]; Bernier, 2020 [12]; Fuentebella, 2025 [5]; Lin, 2025 [4]; Palareti, 2024 [14]; Lim, 2024 [18]; Chen, 2022 [17]; Siedler, 2022 [3]; Mavri, 2021 [9]).

The pooled incidence of thrombotic events across studies was 7% (95% CI: 0.05–0.09, *I*^2^ = 87.3%, *p* < 0.001) (Figure 4). The highest event rates were reported in Fuentebella 2025 [5] (26%, 95% CI: 0.16–0.37), Lim 2024 [18] (21%, 95% CI: 0.13–0.30), and Nguyen 2021 [8] (15%, 95% CI: 0.10–0.21), while the lowest rates were observed in Lin 2025 [4] (3%, 95% CI: 0.02–0.04), Palareti 2024 [14] (4%, 95% CI: 0.03–0.05), and Miklič 2019 [13] (2%, 95% CI: −0.02–0.05).

#### 4.4.5. Meta-Regression Analyses

**Figure 5:**
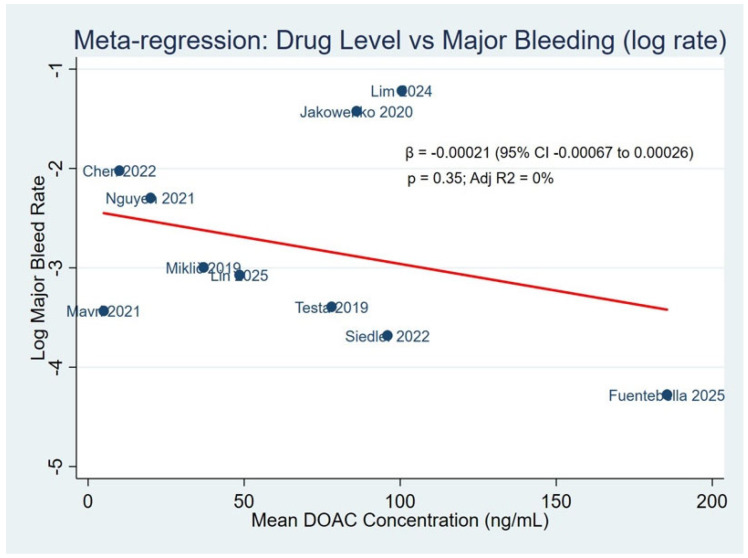
**Meta-Regression: DOAC Concentration vs. Major Bleeding (log rate)** Bubble plot demonstrating the relationship between mean DOAC plasma concentration (ng/mL) and log-transformed major bleeding event rate across studies. The regression slope (red line) was not statistically significant (**β = −0.00021, 95% CI −0.00067 to 0.00026; *p* = 0.35; Adj R^2^ = 0%**), suggesting no meaningful correlation between drug level and bleeding risk. (Jakowenko, 2020 [10]; Nguyen, 2021 [8]; Testa, 2019 [20]; Miklič, 2019 [13]; Fuentebella, 2025 [5]; Lin, 2025 [4]; Lim, 2024 [18]; Chen, 2022 [17]; Siedler, 2022 [3]; Mavri, 2021 [9]).

**Figure 6:**
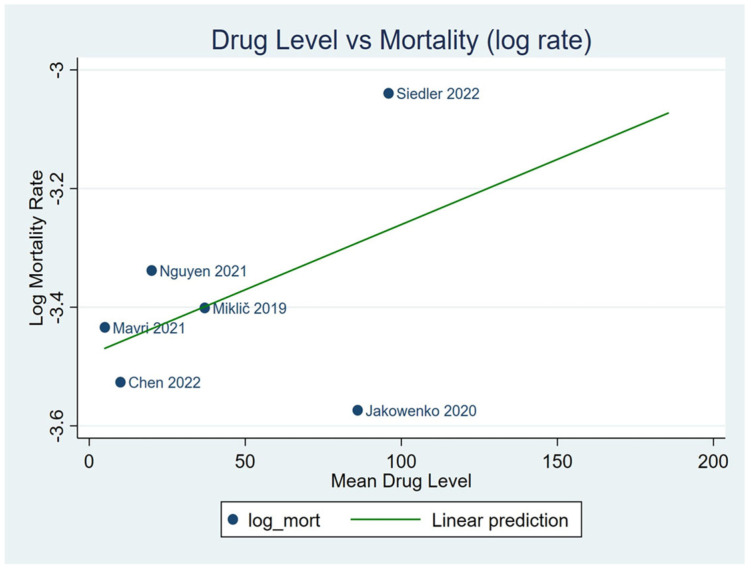
**Meta-regression of drug level vs. mortality.** No clear association was observed between mean DOAC concentration and all-cause mortality (β = 0.00008 [95% CI −0.00033 to 0.00049], *p* = 0.69; Adj R^2^ = 0%). (Jakowenko, 2020 [10]; Nguyen, 2021 [8]; Miklič, 2019 [13]; Chen, 2022 [17]; Siedler, 2022 [3]; Mavri, 2021 [9]).

**Figure 7:**
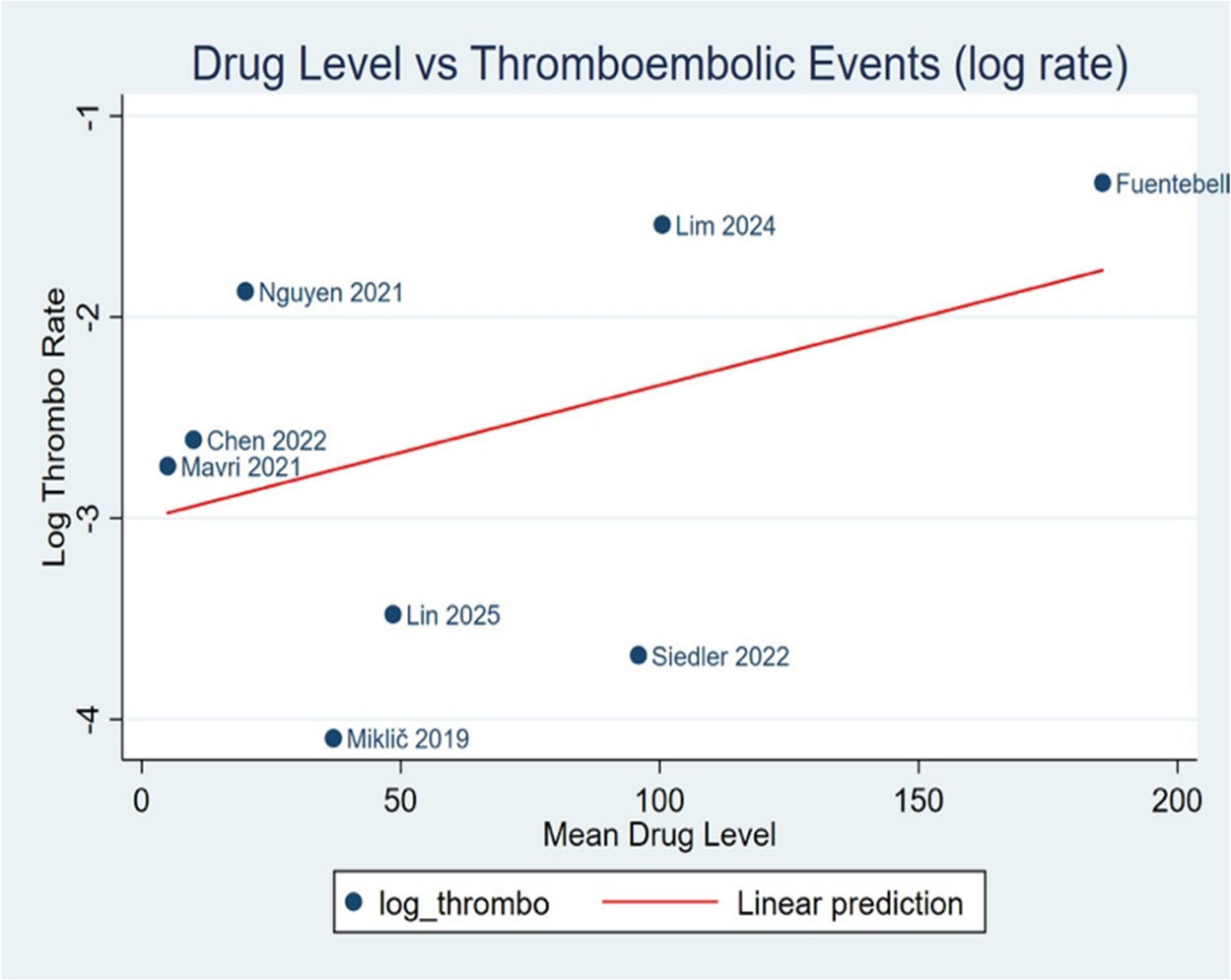
**Meta-Regression: Drug Level vs. Thromboembolic Events (log rate)** Scatter plot showing the relationship between mean direct oral anticoagulant (DOAC) concentration (ng/mL) and log-transformed thromboembolic event rates. The association between drug level and thromboembolic events was not statistically significant (β = 0.00005; 95% CI −0.00041 to 0.00051; *p* = 0.78; Adj R^2^ = 0%). (Nguyen, 2021 [8]; Miklič, 2019 [13]; Fuentebella, 2025 [5]; Lin, 2025 [4]; Lim, 2024 [18]; Chen, 2022 [17]; Siedler, 2022 [3]; Mavri, 2021 [9]).

We conducted meta-regression analysis to evaluate the relationship between mean plasma DOAC concentrations and clinical event rates across the included studies. No statistically significant association was identified between mean concentration and major bleeding (β = –0.00021 [95% CI –0.00067 to 0.00026], *p* = 0.35; Adj R^2^ = 0%) (Figure 5). Although the regression slope was slightly negative, the wide dispersion of data points indicated a weak and inconsistent trend, suggesting that plasma concentration alone does not explain the variability in bleeding risk observed among studies. Further meta-regression analyses insights into thrombotic outcomes. Similarly, no clear association was observed between mean DOAC concentration and all-cause mortality (β = 0.00008 [95% CI −0.00033 to 0.00049], *p* = 0.69; Adj R^2^ = 0%) (Figure 6). An earlier exploratory analysis showed a modest inverse association between mean DOAC concentration and thromboembolic event rates (β = –0.00032 [95% CI −0.00084 to 0.00020], *p* = 0.22; Adj R^2^ = 2%), implying that lower drug exposure tended to coincide with higher thrombotic risk. However, these findings were attenuated and lost significance after applying the more conservative variance correction (Knapp–Hartung adjustment), confirming the absence of a consistent exposure–response relationship for thrombosis across studies (β = 0.00005; 95% CI −0.00041 to 0.00051; *p* = 0.78; Adj R^2^ = 0%) (Figure 7). These findings align with pharmacologic expectations that sub-therapeutic exposure may diminish anticoagulant protection, though the observed trend did not reach statistical significance. The near-zero slope with broad confidence intervals suggests that mortality outcomes are likely driven by non-pharmacokinetic factors—such as comorbidities, underlying indication severity, and competing causes of death—rather than direct drug-level variability. Taken as a whole, these analyses indicate that while biologically plausible concentration–effect trends were observed for both bleeding and thrombotic outcomes, none achieved statistical significance in pooled meta-regression. This underscores the exploratory nature of these findings and highlights the need for standardized, patient-level studies to establish clinically actionable therapeutic ranges for DOAC exposure.

#### 4.4.6. Mortality Rate

**Figure 8:**
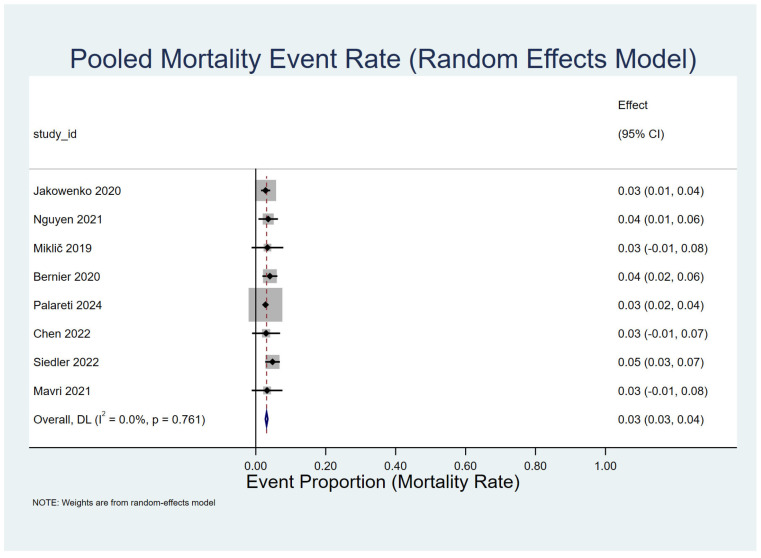
**Pooled Mortality Event Rate (Random-Effects Model)** Forest plot showing study-specific and pooled mortality proportions. The pooled mortality rate was **3% (95% CI: 0.03–0.04)** with negligible heterogeneity (**I^2^ = 0%, *p* = 0.761**). Mortality estimates were consistent across studies, ranging from **3% to 5%**. (Jakowenko, 2020 [10]; Nguyen, 2021 [8]; Miklič, 2019 [13]; Bernier, 2020 [12]; Palareti, 2024 [14]; Chen, 2022 [17]; Siedler, 2022 [3]; Mavri, 2021 [9]).

**Figure 9:**
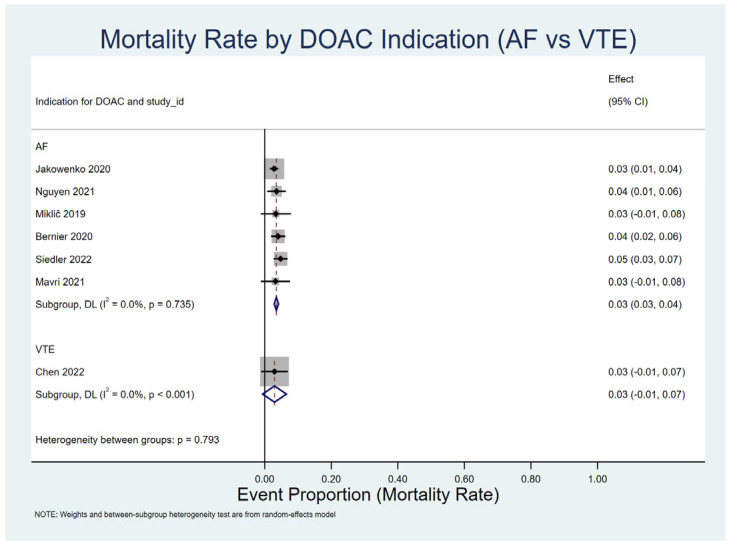
**Mortality Rate by DOAC Indication (AF vs. VTE)** Subgroup analysis comparing mortality rates by clinical indication. Among atrial fibrillation studies, the pooled mortality rate was **3% (95% CI: 0.03–0.04; I^2^ = 0%)**, while venous thromboembolism studies showed a similar rate (**3%, 95% CI: −0.01 to 0.07; I^2^ = 0%)**. Between-group heterogeneity was non-significant (***p* = 0.79**). (Jakowenko, 2020 [10]; Nguyen, 2021 [8]; Miklič, 2019 [13]; Bernier, 2020 [12]; Chen, 2022 [17]; Siedler, 2022 [3]; Mavri, 2021 [9]).

The pooled mortality rate across all studies was 3% (95% CI: 0.03–0.04), with negligible heterogeneity (*I*^2^ = 0.0%, *p* = 0.761) (Figure 8 and Figure 9). Mortality proportions were consistently low across studies, ranging from 3 to 5%, with Bernier 2020 [12] and Siedler 2022 [3] reporting rates near the upper end of this range.

## 5. Discussion

This systematic review and meta-analysis of 19 studies involving 5770 patients evaluated the association between measured plasma concentrations of DOACs and clinical outcomes, including bleeding, thrombotic events, and mortality. The pooled analysis demonstrated an overall major bleeding rate of approximately 8% (95% CI 0.05–0.11) and a thromboembolic event rate of 7% (95% CI 0.05–0.09), with a pooled mortality rate of 3%. These estimates are higher than those reported in landmark randomized controlled trials of DOACs, such as RE-LY and ARISTOTLE, where annual major bleeding and thrombotic event rates were typically in the range of 2–3% and 1–3%, respectively [21,22]. The discrepancy likely reflects the inclusion of real-world, high-risk patient populations undergoing therapeutic drug monitoring (TDM), including those with renal impairment, extremes of body weight, and polypharmacy; groups typically underrepresented in pivotal trials. While earlier analyses suggested a strong exposure–response relationship, our meta-regression found no statistically significant association between mean DOAC concentration and either major bleeding (β = −0.00021, *p* = 0.35 Adj R^2^ = 0%), or thromboembolic events (b = 0.00005, *p* = 0.78), with adjusted R^2^ values of 0%. This indicates that inter-study variability is likely explained by other factors, particularly differences in drug type, indication, and analytical assay methods, rather than plasma concentration alone. The results should therefore be interpreted as exploratory rather than confirmatory.

The observed bleeding and thrombotic event rates broadly align with previously reported estimates from clinical and pharmacokinetic investigations. Testa et al. (2018) demonstrated that subtherapeutic trough DOAC levels were associated with higher thromboembolic risk in atrial fibrillation (AF) patients, supporting the biological plausibility of our findings [11]. Previous studies such as Miklič (2019) [13] and Siedler (2022) similarly reported increased thrombotic risks among patients with lower drug concentrations [3]. These results emphasize that inadequate anticoagulant exposure—whether from underdosing, impaired absorption, or pharmacokinetic variability—may predispose to thrombotic complications, even though the strength of evidence remains limited by between-study heterogeneity.

Conversely, supratherapeutic exposure was associated with a trend toward higher bleeding risk, although there was instability of the point estimate on meta-regression analyses. This aligns with pharmacodynamic expectations and prior data suggesting that elevated DOAC concentrations increase hemorrhagic risk. Nevertheless, the absence of a robust linear relationship across pooled studies may reflect threshold or nonlinear effects, where bleeding risk increases disproportionately beyond certain plasma concentration cutoffs rather than in a continuous, linear predictable fashion.

Our findings contrast with those of Elshafei et al. [23], whose meta-analysis of nine studies (*n* = 159,514) in patients with AF and low body weight (<60 kg or BMI <18 kg/m^2^) found comparable bleeding and thrombotic event rates to the general population. While both syntheses demonstrate the overall safety of DOACs, our TDM-focused approach specifically interrogates biological plausibility (i.e., how measured exposure relates to outcomes), whereas Elshafei et al. [23] explored clinical outcomes by weight category. Collectively, the evidence suggests that although DOACs remain broadly safe and effective across populations, pharmacokinetic variability likely contributes to individual differences in response and outcomes [27,28,29].

Heterogeneity in this review (*I*^2^ > 90%) stems from multiple interrelated sources. Firstly, differences in DOAC type substantially influence pooled results. Dabigatran, a direct thrombin inhibitor with 80% renal excretion, demonstrates wider interindividual variability in patients with renal dysfunction compared to factor Xa inhibitors such as apixaban or rivaroxaban, which undergo combined hepatic and renal clearance. Consequently, studies that pooled different DOACs introduced inherent pharmacokinetic variability.

Secondly, primary DOAC indications contribute to heterogeneity. Patients with atrial fibrillation (AF) typically represent older populations with multiple comorbidities and prolonged treatment durations, whereas venous thromboembolism (VTE) patients may receive shorter courses with different baseline risk profiles. Our pooled estimates showed higher bleeding rates among AF patients (12%) compared to VTE patients (7%), reflecting these demographic and clinical distinctions.

Thirdly, assay methodology represents a major methodological source of heterogeneity. Anti-factor Xa chromogenic assays, commonly used for apixaban, rivaroxaban, and edoxaban, can vary by as much as 30% across laboratories due to differences in calibration and reagent sensitivity. By contrast, studies employing liquid chromatography–tandem mass spectrometry (LC–MS/MS) provide more precise quantification but are resource-intensive and less standardized. Variability in assay selection, calibration curves, and timing of measurement (peak vs. trough) undermines comparability and may obscure true exposure–response relationships.

Furthermore, renal function and polypharmacy are important clinical contributors. Few included studies reported stratified analyses by renal impairment, yet impaired kidney function is a well-established determinant of increased DOAC exposure. Concomitant use of *P-glycoprotein* (P-gp) or *CYP3A4* inhibitors (such as amiodarone, verapamil, and azole antifungals) further modifies DOAC levels, yet most studies lacked standardized reporting of these covariates. Similarly, polypharmacy, extremes of body weight, and hypoalbuminemia may alter free drug fraction and distribution volume, compounding between-study differences.

Finally, regional and ethno-geographic prescribing practices (driven both by economic considerations as well as peculiar local data) influence variability. Asian cohorts, for example, frequently employ lower DOAC doses due to perceived bleeding risk and pharmacogenetic differences, including polymorphisms in drug-metabolizing enzymes [30,31,32]. Such practices, while locally justified, yield systematically lower trough concentrations, potentially compromising efficacy. Conversely, European and North American studies typically use full-dose regimens and advanced LC–MS/MS quantification, introducing methodological rather than biological heterogeneity [33,34,35,36].

The findings of this meta-analysis contribute to the evolving discussion surrounding the “no monitoring needed” paradigm for DOACs. Fixed-dose regimens have been promoted based on predictable pharmacokinetics and wide therapeutic windows. However, the current synthesis challenges this assumption by demonstrating that variability in plasma exposure (though not linearly predictive of outcomes) may still hold clinical relevance in certain subgroups. A targeted monitoring strategy could therefore be considered in patients with characteristics known to influence DOAC pharmacokinetics, such as renal dysfunction, extreme body weight, hepatic impairment, or multiple interacting medications. In such cases, periodic drug-level measurement could guide dose adjustment or confirm therapeutic adequacy, particularly when transitioning between agents or managing recurrent events despite adherence. However, routine universal monitoring is not currently supported by the available data.

Moreover, the absence of clear thresholds linking plasma concentration to clinical outcomes underscores the need for standardized TDM protocols. Harmonization of sampling time (trough vs. peak), assay calibration, and reporting standards would enhance comparability and facilitate individualized dosing models. As evidence accumulates, integration of TDM into clinical decision support systems—particularly within high-risk populations—could improve the balance between efficacy and safety. These results also suggest that heterogeneity in reported outcomes across trials may partly reflect underlying population differences rather than inconsistencies in drug performance. Thus, clinicians should interpret DOAC concentrations within the clinical context, considering factors such as renal function, age, concomitant medications, and bleeding risk profiles rather than applying fixed cutoffs.

Emerging data relating to PK/PD modeling suggest that the relationship between DOAC plasma concentration and proven clinical outcomes may be nonlinear (rather than proportional). For several agents (including dabigatran, apixaban, and rivaroxaban), population modeling and anti–Xa activity curves indicate a plateau of anticoagulant effect within the usual therapeutic range, with disproportionately increased bleeding risk once concentrations exceed an upper inflection point [19,37,38]. Conversely, thromboembolic risk appears to increase sharply only at very low trough levels, particularly below the lower decile of exposure reported in some observational cohorts [25]. Our findings similarly align with the hypothesis of a U-shaped or threshold-based exposure–response curve, in which risk concentrates at the extremes rather than across the full range of drug concentrations. Another implication relates to existing “expected” or “on-therapy” DOAC plasma concentration ranges. These ranges, including those summarized by de Vries et al. (2025) [25], represent descriptive population percentiles, not validated therapeutic targets. They were derived from studies with varying designs and differences in sampling time, kidney function, assay methodology, and co-medications. No DOAC currently has an outcome-validated therapeutic window analogous to that used for vitamin-K antagonist (INR). Therefore, while our findings show that adverse events tend to cluster among patients with concentrations far outside these published ranges, they do not establish that these ranges confer clinical safety or efficacy. Instead, our results underscore the need for prospective, outcome-based PK/PD studies to determine whether meaningful therapeutic windows can be defined for selected high-risk populations.

## 6. Strengths and Limitations

The present study possesses several strengths. It represents the most comprehensive quantitative synthesis of secondary data on DOAC therapeutic drug monitoring (TDM) to date, employing robust search strategies, rigorous inclusion criteria, and adherence to PRISMA and MOOSE guidelines. The inclusion of both anti–Factor Xa–based and LC–MS/MS–based studies enables cross-validation of findings across different assay platforms, while the use of meta-regression allows exploration of potential effect modifiers. The consistency of pooled event estimates across independent analytical methods strengthens the internal validity of our results.

Nonetheless, several limitations must be acknowledged. A key limitation relates to assay variability. Some included studies utilized non-drug-specific anti–Xa chromogenic assays, which can differ significantly in calibration, sensitivity, and inter-laboratory reproducibility. These assays were often not calibrated to the DOAC of interest (e.g., apixaban or rivaroxaban), which may introduce measurement bias and limit the comparability of plasma concentrations across studies. Second, the lack of standardized therapeutic ranges for DOACs remains a major barrier to interpreting plasma concentration data. Reported “on-therapy” ranges are based on descriptive population percentiles and were not derived from outcome-validated thresholds. Therefore, observed plasma levels outside these ranges cannot be reliably classified as subtherapeutic or supratherapeutic in the absence of validated targets. Third, confounding by indication and patient-level characteristics is an inherent limitation of this meta-analysis. Most included studies were observational and often enrolled high-risk populations, such as elderly patients, individuals with renal impairment, or those with multiple comorbidities. It is possible that TDM was selectively performed in these complex cases, introducing selection bias and reducing generalizability to the broader population of DOAC users. The absence of subgroup analyses stratified by age, renal function, or comorbidity burden further limits our ability to disentangle pharmacokinetic variability from underlying clinical risk.

High statistical heterogeneity (*I*^2^ > 90%) was observed across outcomes, driven by differences in DOAC type, assay method, timing of sample collection (peak vs. trough), and patient demographics. Although random-effects models and meta-regression were employed to mitigate these effects, the residual variability highlights the need for individual patient–level data and standardized protocols. In addition, small-sample studies may have inflated variance, particularly those from single-center cohorts. Publication bias cannot be excluded, as studies demonstrating null or negative correlations between drug levels and outcomes may be underrepresented in the literature. Finally, mortality outcomes were inconsistently reported and lacked standardized definitions, limiting the interpretation of pooled mortality estimates.

Despite these limitations, the convergence of evidence across multiple analytical approaches suggests that variability in DOAC exposure remains clinically relevant and may account for some residual risk of adverse outcomes in high-risk subgroups. These insights provide a foundation for future prospective studies and reinforce the importance of pharmacokinetic stratification in precision anticoagulation.

## 7. Conclusions

In this systematic review and meta-analysis, measured DOAC concentrations show a limited and inconsistent association with clinical outcomes. While the present synthesis does not demonstrate a statistically robust linear correlation between DOAC plasma concentrations and adverse outcomes, it highlights the multifactorial determinants of bleeding and thrombosis risk, underscores the potential value of selective TDM in individualized care. Further prospective, standardized studies are needed to define clinically actionable thresholds and to validate TDM-guided strategies that optimize the delicate balance between safety and efficacy in DOAC therapy.

## Figures and Tables

**Table 1:** Study Characteristics.

Study ID	Country	Study Design	Sample Size (*n*)	Indication	DOAC(s)	Assay Method	TDM Threshold	Age (Years)	Gender % Male	Thrombotic Events (*n*)	Bleeding Events (*n*)
**Jakowenko, 2020** [10]	USA	Retro. cohort	606	AF, DVT, PE, Other	API, RIV	Anti-Xa assay	-	72 (IQR 61–81)	48%	-	146
**Nguyen, 2021** [8]	USA	Retro. descrip.	169	AF, DVT, PE, Other	API, RIV	Anti-Xa assay	-	69 (IQR 56–79)	51%	26	17
**Testa, 2018** [11]	Italy	Retro. cohort	565	NVAF	API, RIV, DAB	dTT; anti-FIIa; anti-FXa	Quartiles (I-IV)	80 (IQR 44–97)	55.80%	10	-
**Testa, 2019** [20]	Italy	Retro. cohort	565	NVAF	API, RIV, DAB	dTT; anti-FIIa; anti-FXa	Quartiles (I-IV)	80 (IQR 44–97)	55.80%	-	19
**Miklič, 2019** [13]	Slovenia	Pros. cohort	60	AF	RIV	LC-MS/MS; Anti-Xa; PT & APTT	50 ng/mL (for trough levels)	73 ± 7	53.30%	1	3
**Bernier, 2020** [12]	France	Pros. cohort	322	AF	DAB, RIV	HPLC-MS/MS	5th/95th percentile (pivotal trials)	78.5 ± 13.1	52.00%	26	44
**Fuentebella, 2025** [5]	USA	Retro. cohort	72	AF, DVT, PE	API	-	-	67.5 (IQR 60–71)	64%	19	1
**Lin, 2025** [4]	Tawain	Pros. cohort	1037	AF	RIV, API, EDO	UHPLC–MS/MS	RIV: 12–137 ng/mL; API: 34–230 ng/mL; EDO: 12–43 ng/mL	75.4 ± 10.0	53.70%	32	48
**Stretton, 2025** [6]	Australia	Retro. cohort	86	AF, VTE	API, RIV, DAB	Anti-Xa assay	-	76.7 ±9.3	64.10%	-	86
**Al-Aieshy, 2024** [7]	Sweden	Cross-sectional	85	AF	API	LC-MS/MS	>100 mcg/L	3 groups (range 44–86)	37.64%	-	-
**Zhang, 2024** [15]	China	Pros. cohort	112	CVT	RIV	Anti-Xa assay	0.3–0.7 IU	41.2 ± 15.6	39.29%	-	11
**Palareti, 2024** [14]	Italy	Pros. cohort	1657	AF	API, DAB, EDO, RIV	Anti-Xa assay	3 classes of standardized C-trough values	80 (47–100)	45.90%	71	30
**Lim, 2024** [18]	Australia	Retro. cohort	98	AF, VTE, Other	API, RIV, DAB	Anti-Xa assay	30 & 50 ng/m (ISTH guidelines)	71.7 ± 14.5	64.2%	21	29
**Bozic, 2023** [16]	Croatia	Pros. observ.	90	AF, DVT, PE, Other	DAB, API, RIV	Anti-Xa assay	Literature-based Cmax & Ctrough	78.8 ± 8.3	54.4%	-	90
**Lin, 2023** [2]	Taiwan	Pros. observ.	138	AF, VTE	DAB, RIV, API, EDO	UHPLC-MS/MS	<50 ng/mL	IS group: 75.7 ± 12.1; ICH group: 77.8 ± 9.3 years	51.4%	-	-
**Chen, 2022** [17]	USA	Retro. descrip.	68	VTE	API	Anti-Xa assay	-	61 ± 16	25%	5	9
**Siedler, 2022** [3]	Germany	Observ. cohort	397	2° prevention of IS in AF	API, DAB, EDO, RIV	Anti-Xa assay	-	78 ± 9	49.4%	10	10
**Ballerie, 2021** [1]	France	Pros. observ.	146	VTE	API, RIV	Anti-Xa assay	-	61 (19–86)	52%	2	-
**Mavri, 2021** [9]	Slovenia	Pros. observ.	62	AF	API	LC–MS/MS; anti-Xa assay	-	78 ± 8 years	40.3%	4	2

API = Apixaban; DAB = Dabigatran; EDO = Edoxaban; AF = Atrial fibrillation; NVAF = Non valvular atrial fibrillation; VTE = Venous Thromboembolism; PE = Pulmonary embolism; DVT = Deep vein thrombosis; CVT = Cerebral venous thrombosis; ISTH = International Society of Thrombosis & Haemostasis; IS = Ischemic stroke; ICH = Intracranial hemorrhage.

## Data Availability

Available from the corresponding author on reasonable request.

## Supplementary Materials

The following supporting information can be downloaded at: https://www.mdpi.com/article/10.3390/ph19020215/s1, Table S1. Full-string search strategy of Medline database; Table S2. Newcastle–Ottawa Scores, Risk of Bias (RoB), and Study Limitations.
