# Supplementary material for: Therapeutic Drug Monitoring of Direct Oral Anticoagulants and Its Association with Clinical Outcomes: A Systematic Review and Meta-Analysis
Source: Pharmaceuticals (Basel). 2026 Jan 26;19(2):215. doi: 10.3390/ph19020215 (PMC12943562; doi:10.3390/ph19020215)
Supplement: Supplementary file 1 [file pharmaceuticals-19-00215-s001.zip › pharmaceuticals-3994395-supplementary.pdf]

**Supplementary Table S1: Full-string search strategy of Medline database**
(("Direct Oral Anticoagulants"[Mesh] OR "direct oral anticoagulant*"[tiab] OR "DOAC"[tiab] OR "dabigatran"[tiab] OR "rivaroxaban"[tiab] OR "apixaban"[tiab] OR "edoxaban"[tiab]))<br>AND<br>(("Therapeutic Drug Monitoring"[Mesh] OR "drug concentration*"[tiab] OR "plasma concentration"[tiab] OR "pharmacokinetic*"[tiab]))<br>AND<br>(("Hemorrhage"[Mesh] OR bleeding[tiab] OR hemorrhag*[tiab] OR "Thrombosis"[Mesh] OR thrombos*[tiab] OR "Stroke"[Mesh] OR "Venous Thromboembolism"[Mesh] OR VTE[tiab]))

Supplementary Table S2. Newcastle–Ottawa Scores, Risk of Bias (RoB), and Study Limitations.

Study ID	NOS Score (max 9)	Risk of Bias Summary	Narrative Description of Limitations
Fuentebella, 2025	4	critical	Retrospective, selected group (lung transplant), confounding by drug interaction e.g.: antifungal and immunosuppressives.
Lin, 2025	7	moderate	single trough measurement, single-centered and lack of diversity, no peak level assessment
Stretton, 2025	4	critical	Retrospective and single-centered, small sample, doesn't use globally validated TDM assay.
Al-Aieshy, 2024	5	serious	cross-sectional measuring single drug concentration, no bleeding or thrombotic events.
Zhang, 2024	6	serious	single-centered, no randomization of subgroups
Palareti, 2024	7	moderate	Single-time measurement, impact of COVID-19
Jakowenko, 2020	5	serious	Retrospective with inclusion of known bleeding events, single-center limited generalizability.
Nguyen, 2021	5	serious	Retrospective and single-centered, small sample.
Testa, 2018	8	low	Retrospective, single measurement of DOAC level, adherence uncertainty as follow-up is after 1 year, confounded by high CHA_2DS_2-VASc scores.
Testa, 2019	8	low	Retrospective, single measurement, timing, and adherence uncertainty.
Miklič, 2019	7	moderate	Small sample, all patients from the anticoagulation clinic, single measurement of drug level.
Bernier, 2020	8	low	Dose is not reported, high risk patients (high CHA_2DS_2-VASc)
Lim, 2024	5	serious	Retrospective, single center, single measurement
Bozic, 2023	5	critical	Single center, no serial measurements.
Lin, 2023	7	moderate	Single measurement, Dose is not reported, no adjustment for stroke subtype and infarct size
Chen, 2022	3	critical	Specific population (ESRD), small sample, no follow-up.
Siedler, 2022	8	low	Single center, old population with high CHA_2DS_2-VASc score,
Ballerie, 2021	7	moderate	Single measurement, obese patients,
Mavri, 2021	6	serious	Old patients, small sample, COVID-19 impact (more adherence)

NOS = Newcastle-Ottawa scale; NRS = non-randomized study.
